# U.S. EPA Reference Standards and Quality Assurance Materials for the Analysis of Environmental Pollutants

**DOI:** 10.6028/jres.093.028

**Published:** 1988-06-01

**Authors:** R. E. Thompson, J. R. Tuschall, T. M. Anderson

**Affiliations:** Northrop Services, Inc.—Environmental Sciences, Research Triangle Park, NC 27709

“The quality of all analytical measurements rests ultimately upon the quality of reference materials employed.” This statement is especially appropriate in the determination of trace contaminant residues in environmental matrices. To support the need for a certified, quality-controlled, common database in the analysis of pollutant chemical agents in environmental substrates, the U.S. EPA’s Environmental Monitoring Systems Laboratory (Las Vegas) and Environmental Monitoring and Support Laboratory (Cincinnati) jointly maintain several repositories of analytical grade reference materials under the Agency’s Quality Assurance Reference Materials (QARM) Project. Operated under contract by Northrop Services in Research Triangle Park, NC, the Project currently offers standards of some 2700 compounds of environmental concern, including pesticides and their metabolites and degradation products; PCBs, chloro- and bromodibenzodioxins and furans, and other halogenated organics; plasticizers; nitrosamines; polynuclear aromatics; and heavy metals and metalloids. Included among these are the Agency-identified compounds commonly known as the “priority pollutants,” those materials regulated under Appendix VIII (RCRA) and CERCLA (Superfund) legislation, and groundwater monitoring compounds.

The program is currently being supplied by nearly 200 chemical manufacturing companies and 33 chemical supply houses. Additional commercially-unavailable compounds, especially select environmental reaction and degradation products, are synthesized and purified in-house. Standards are offered, without charge, to any and all laboratories worldwide engaged in environmental monitoring, research, and/or regulatory and enforcement activities. During 1986, more than 150,000 standards were distributed in response to 7,000 requests.

The analyst using Project standards expects the highest quality materials at accurate purity values and concentrations (for solutions), often without knowledge of how the integrity of each is established and maintained. Prior to distribution, each compound is subjected to a rigorous quality assurance program, including (1) component identification, (2) purity assay of neat materials, (3) concentration verification of solutions, and (4) stability studies, using a variety of instrumental methods.

Although acquired materials typically include a percent purity value, each is individually reanalyzed in a series of steps. Chemical identity is verified by mass spectrometry, introduced via gas chromatograph or alternately, by direct insertion probe. For select compounds, other spectroscopic methods—IR, NMR, and ultraviolet spectroscopy—are supplementally employed.

Subsequently, chemical purity is assayed by (at least) two methods, normally one chromatographic (GC, HPLC, TLC) and one non-chromatographic (DSC, NMR, IR, etc.). Establishing purity of a neat material is often complicated by the lack of a uniform response for all components to a single mode of detection. Electron capture, flame ionization, nitrogen-phosphorus, Hall, or mass spectrometric detection are the chromatographic methods of choice for most compounds, whereas HPLC or TLC are used for highly polar or thermo-labile materials. Purity is established by the ratio of peak area of the compound of interest to the combined peak areas for all eluting components. Implicit in this procedure is the assumption that all components elute and produce equal response at the detector. For FID or MS (positive electron impact), the assumption of equal responses is reasonably accurate, although some differences in relative response are inevitable.

Non-chromatographic methods of purity analysis include ultraviolet spectroscopy, NMR, melting point, and differential scanning calorimetry (DSC). The latter method is frequently used for high-purity compounds because the method is typically reliable at purities >97% and useful for a wide variety of compounds. In DSC analysis, purity is estimated using a van’t Hoff plot of melting point data. Typically, agreement of results among methods is within 1–2% and an average value is reported. However, in some cases, a substantial discrepancy in results is observed, necessitating further analyses. Additionally, isotopic purity of compounds enriched with stable isotopes (^13^C, ^37^Cl, D) is established by mass spectrometry. Typical purity of materials distributed exceeds 99%.

The two other components of the quality assurance program that apply to materials available in solution form are concentration verification and stability studies. Subsequent to loading 1.5 mL of a prepared standard solution into several thousand ampuls, a random sample is analyzed from each of three groups (early, middle, and late) of ampuls filled. Analysis (in-house as well as by external referee laboratories) by GC, HPLC, or ultraviolet spectroscopy is performed to establish that concentrations of solutions are within 10% of the target value. Similarly, stability studies (again, on randomly selected ampuls) are performed at predetermined intervals for the duration of storage to verify concentration for prepared solutions in each solvent used. Any compound determined to be unstable is freshly prepared and again subjected to the entire QA program prior to distribution.

In addition to analytical standards, the program supports laboratory quality assurance efforts with other services. Florisil, a differential adsorbent useful in sample clean-up and multiresidue extractions, is available, as are several pre-coated GLC column packings particularly suitable for environmental analysis. Each batch is screened and certified for acceptable separation and recovery, and each sample is provided with an accompanying performance specification sheet and elution profile.

Four times per year, under the Intercomparison Studies project, “spiked” matrix reference materials are provided to laboratories electing to participate in EPA’s interlaboratory quality assurance program. Porcine fat, human blood plasma, human urine, and water-miscible solvents are custom-spiked with multi-compound residue levels of various compounds. Field laboratories analyze these materials and return results to be graded in an effort to assess instrumentation accuracy and precision as well as analytical techniques. These samples typically serve a dual purpose as (1) check samples of unknown composition for laboratory intercomparison and (2) after composition is disclosed, internal laboratory control samples for the next 3–6 months.

Technical manuals on analytical methods, analytical quality control, standards preparation, and related topics are also available. An extensive technical library is maintained for requestors’ needs, complete with direct terminal access to several major computerized chemical databases. Technical information specialists provide assistance to laboratories with chemical/physical/toxicological data; sample preparation and analytical methodology; instrument troubleshooting; synthesis and purification methods; quality assurance procedures; transportation and waste disposal protocols; and a wide variety of related information. More than 4,000 environmental monitoring and research laboratories in 93 countries are currently utilizing program services.

